# Levothyroxine Replacement Alleviates Thyroid Destruction in Hypothyroid Patients With Autoimmune Thyroiditis: Evidence From a Thyroid MRI Study

**DOI:** 10.3389/fendo.2019.00138

**Published:** 2019-03-11

**Authors:** Jia Liu, Zhe Chen, Min Liu, Yumei Jia, Zhi Yao, Guang Wang

**Affiliations:** ^1^Department of Endocrinology, Beijing Chao-Yang Hospital, Capital Medical University, Beijing, China; ^2^Department of Radiology, China-Japan Friendship Hospital, Beijing, China

**Keywords:** autoimmune thyroiditis, hypothyroidism, levothyroxine treatment, T1-mapping, thyroid destruction

## Abstract

**Background:** Autoimmune thyroiditis (AIT) is the most frequent cause of hypothyroidism. Our previous studies have shown that magnetic resonance T1-mapping is a new technique for quantitatively evaluating the degree of thyroid destruction in AIT patients. This study aimed to evaluate the effect of levothyroxine on thyroid destruction in hypothyroid AIT patients using thyroid T1-mapping technique.

**Methods:** This study recruited 29 hypothyroid AIT patients and 18 age- and sex-matched healthy individuals. Thyroid T1-mapping values were measured in all participants and repeated in the AIT patients at 3 months after they achieved a euthyroid state following levothyroxine treatment.

**Results:** Thyroid T1-mapping values were higher in the AIT patients than in the healthy controls (1167.2 ± 163.2 vs. 779.6 ± 83.8 ms, *P* < 0.01), and levothyroxine treatment significantly decreased the thyroid T1-mapping values of AIT patients (1006.3 ± 114.6 vs. 1167.2 ± 163.2 ms, *P* < 0.01). Meanwhile, the reduced levels of anti-peroxidase antibody (TPOAb) and anti-thyroglobulin antibody (TgAb) were observed in the AIT patients after levothyroxine treatment [TPOAb: 257.6 (23.9–960.6) vs. 1,287.4 (12.6–2000.0) IU/mL, *P* < 0.01; TgAb: 53.54 (9.58–386.2) vs. 103.9 (34.2–1,596.8) IU/mL, *P* < 0.05]. High-sensitivity C-reactive protein (hsCRP) levels showed a descending tendency following levothyroxine treatment, although there was no statistical difference (*P* > 0.05).

**Conclusions:** In the AIT patients, thyroid T1-mapping values were significantly increased, and levothyroxine treatment significantly decreased the thyroid T1-mapping values of the AIT patients. These results might suggest that levothyroxine treatment alleviates thyroid destruction in hypothyroid AIT patients.

## Introduction

Thyroid hormone plays a pivotal role in regulating energy metabolism and organ function (1). Hypothyroidism is one of the most common endocrine diseases and causes multiple metabolic disorders and tissue damage (2, 3). Untreated hypothyroid patients may present with various clinical signs, including cognitive disorders, dyslipidemia, heart failure, psychosis, and infertility (2, 3). The most frequent cause of hypothyroidism is autoimmune thyroiditis (AIT), which is an organ-specific autoimmune disease characterized by diffuse lymphocytic infiltration, fibrosis, and epithelial cell destruction (2, 4). Levothyroxine replacement is the main therapy for hypothyroidism (2). Levothyroxine treatment ameliorated metabolic disorders, restored functions of heart and skeletal muscle, and improved cognitive performance in patients with hypothyroidism (2, 5, 6). Our recent studies showed that short-term levothyroxine treatment reverses diffuse myocardial injuries in hypothyroid AIT patients (6). However, it remains unclear whether levothyroxine treatment can alleviate thyroid destruction in hypothyroid AIT patients.

Thyroid longitudinal relaxation time mapping (T1-mapping) measured by magnetic resonance imaging is a parametric reconstructed image that has been considered as a new technique for evaluating tissue fibrosis and edema (7–10). Increased T1-mapping values were associated with fibrosis degree in histology (9, 11). Meanwhile, T1-mapping values were also affected by water molecules in the tissue, which suggested that tissue edema, like fibrosis, increases T1-mapping values (12, 13). Our previous study showed that thyroid T1-mapping quantitatively evaluates the degree of thyroid destruction in AIT patients (7). This study aimed to evaluate the effect of levothyroxine on thyroid destruction in hypothyroid AIT patients using thyroid T1-mapping technique.

## Materials and Methods

### Study Design and Participants

A total of 29 drug-naïve AIT patients with overt hypothyroidism were recruited from the Endocrinology Department of Beijing Chao-Yang Hospital between March 2014 and March 2015. Meanwhile, 18 age- and sex-matched healthy individuals without AIT were enrolled as the healthy control group. Free T3 (FT3), free T4 (FT4), thyroid-stimulating hormone (TSH), anti-peroxidase antibody (TPOAb), anti-thyroglobulin antibody (TgAb), and thyroid ultrasound were performed in all participants. The healthy control subjects were defined based on the following criteria: serum levels of FT3, FT4, and TSH were in the normal ranges (FT3: 2.63–5.71 pmol/L; FT4: 9.10–19.24 pmol/L; TSH: 0.35–4.94 mIU/L); both TPOAb and TgAb were negative; and thyroid ultrasound was normal. Overt hypothyroidism was diagnosed as increased serum TSH levels and decreased FT4 levels. AIT was diagnosed by elevated TPOAb (reference range: 0.00–60.00 IU/mL) and/or TgAb (reference range: 0.00–60.00 IU/mL) and diffuse thyroid enlargement with typical hypoechoic or heterogeneous thyroid in a thyroid ultrasound (14). No participants had systemic inflammatory disease, cancer, metal implants, or claustrophobia. Individuals who were pregnant, possibly pregnant, or ingesting agents known to influence thyroid function were also excluded. All of the hypothyroid patients were given levothyroxine treatment (Levothyroxine Sodium Tablets, Merck). Levothyroxine was administrated orally in a single dose in the morning between 30 and 60 min before breakfast. The initial dose was 50 μg/d, and thyroid function was measured every 4 weeks for dose adjustment. The levothyroxine dose was adjusted by 25 μg each time until the euthyroid state was achieved (FT4: 9.10–19.24 pmol/L; TSH: 0.35–4.94 mIU/L). All enrolled subjects provided written informed consent. This study's protocol was approved by the Ethics Committee of the Beijing Chao-Yang Hospital, Capital Medical University.

### Clinical Tests

Information about each patient's health status and medications was collected using a standard questionnaire. Height and weight were measured to the nearest 0.1 cm and 0.1 kg, respectively, by the same trained group. Body mass index (BMI) was calculated as the weight in kilograms divided by the height in meters squared. Blood samples were obtained after overnight fasting and stored at −80°C. FT3, FT4, and TSH levels were measured by electrochemiluminescence immunoassay using Abbott Architect i2000 (Abbott Diagnostics, Abbott Park, IL, USA). The serum concentrations of TgAb and TPOAb were detected by chemiluminescent immunoassay. High-sensitivity C-reactive protein (hsCRP) was measured using an immunonephelometric assay. The thyroid ultrasound was assessed by a well-trained ultrasound physician. In the hypothyroidism group, these measurements were repeated at 3 months after the euthyroid state was achieved.

### Thyroid Magnetic Resonance Imaging

Thyroid magnetic resonance imaging was performed in all participants in a supine position using 3 Tesla scanners on a Tim Trio System (Siemens Healthcare, Erlangen, Germany). Cine images were acquired by gapless whole thyroid coverage, and slice thickness for cine was 8 mm. All routine imaging and maps were analyzed using Argus (SYNGO MMWP Workstation, Siemens AG). T1-mapping was measured by a modified look-locker inversion-recovery (MOLLI) sequence without contrast administration (7, 15). Circle regions of interest were drawn to thyroid tissue in all participants. Shimming and center frequency adjustments were performed to generate off-resonance artifact-free images. Two expert radiologists (M. L., 11 years of experience; F.F.Y., 4 years of experience) were blinded to the groups and performed image analyses for T1-mapping value. The T1-mapping values of the left (T1-L-Thyroid) and right (T1-R-Thyroid) thyroid lobes were calculated by an average across all of the available slices. The final thyroid T1-mapping value (T1-Thyroid) was determined by averaging the values of T1-L-Thyroid and T1-R-Thyroid. In the hypothyroid AIT patients, thyroid magnetic resonance imaging was repeated 3 months after the euthyroid state was achieved.

### Statistical Analysis

Data were analyzed using SPSS 21.0 (SPSS, Chicago, IL, USA). Normally distributed variables were expressed as mean ± standard deviation (SD). Because TSH, TPOAb, TgAb, and hsCRP did not have a normal distribution, they were expressed as medians with upper and lower quartiles. The differences between the control and hypothyroidism groups were analyzed by independent Student's *t*-test or the Mann–Whitney *U*-test. Changes in parameters from baseline values within a group were evaluated using a two-tailed paired *t*-test. All statistical tests are two-tailed, with *P* < 0.05 considered significant.

## Results

### Baseline Characteristics of the Control and Overt Hypothyroidism Groups

The baseline characteristics of the control and overt hypothyroidism groups are summarized in Table 1. There was no significant difference in age or gender between the two groups. The hypothyroid patients had significantly higher body weight and BMI than the control subjects (body weight: 68.4 ± 11.3 vs. 59.2 ± 10.2 kg, *P* < 0.05; BMI: 26.0 ± 3.9 vs. 22.0 ± 3.2 kg/m^2^, *P* < 0.01; Table 1). Significantly increased TSH levels and decreased FT3 and FT4 levels were observed in the hypothyroidism group as compared with the control group [TSH: 100.00 (93.11–100.00) vs. 1.88 (1.41–3.08) mIU/L; FT3: 2.45 ± 0.78 vs. 4.36 ± 0.62 pmol/L; FT4: 5.79 ± 1.54 vs. 14.14 ± 1.63 pmol/L; all *P* < 0.01; Table 1]. We also found higher TPOAb and TgAb titers in the hypothyroid patients compared with the controls [TPOAb: 1287.4 (12.6–2000.0) vs. 0.4 (0.2–0.6) IU/mL, *P* < 0.01; TgAb: 103.9 (34.2–1596.8) vs. 1.3 (0.8–35.9) IU/mL, *P* < 0.05; Table 1]. The hsCRP levels were increased in the hypothyroid patients compared with the control group [0.60 (0.07–1.48) vs. 0.00 (0.00–0.04) mg/L, *P* < 0.01; Table 1]. The hypothyroidism group had significantly higher thyroid T1-mapping values than the control group (1167.2 ± 163.2 vs. 779.6 ± 83.8 ms; *P* < 0.01; Table 1).

**Table 1:** The clinical characteristics of the control and overt hypothyroidism groups.

**Parameters**	**Control group (*n* = 18)**	**Overt hypothyroidism group (*n* = 29)**		***P*_1_**	***P*_2_**	***P*_3_**
		**Baseline**	**After levothyroxine**			
Age,y	35.1 ± 8.1	36.7 ± 9.3		0.489	–	0.489
Gender, Males/Females, *n*	2/16	3/26		0.619	–	0.619
Body Weight, kg	59.2 ± 10.2	68.4 ± 11.3	64.0 ± 10.7	0.010	0.000	0.185
BMI, kg/m^2^	22.0 ± 3.2	26.0 ± 3.9	24.5 ± 4.1	0.001	0.000	0.044
FT3, pmol/L	4.36 ± 0.62	2.45 ± 0.78	4.37 ± 0.62	0.000	0.000	0.871
FT4, pmol/L	14.14 ± 1.63	5.79 ± 1.54	15.19 ± 1.93	0.000	0.000	0.105
TSH, mIU/L	1.88 (1.41–3.08)	100.00 (93.11–100.00)	1.52 (0.81–2.60)	0.000	0.000	0.233
TPOAb, IU/mL	0.4 (0.2–0.6)	1287.4 (12.6–2000.0)	257.6 (23.9–960.6)	0.000	0.006	0.000
TgAb, IU/mL	1.3 (0.8–35.9)	103.9 (34.2–1596.8)	53.54 (9.58–386.2)	0.000	0.027	0.000
hsCRP, mg/L	0.00 (0.00–0.04)	0.60 (0.07–1.48)	0.34 (0.02–0.92)	0.006	0.064	0.022
T1-L-Thyroid, ms	778.2 ± 84.4	1171.2 ± 162.6	1004.0 ± 130.7	0.000	0.000	0.000
T1-R-Thyroid, ms	781.7 ± 83.2	1159.7 ± 169.9	1005.9 ± 103.0	0.000	0.000	0.000
T1-Thyroid, ms	779.6 ± 83.8	1167.2 ± 163.2	1006.3 ± 114.6	0.000	0.000	0.000

*Data are means ± SD unless indicated otherwise. TSH, TPOAb, TgAb, and hsCRP are shown as median, upper, and lower quartiles. BMI, body mass index; FT3, free T3; FT4, free T4; TPOAb, antithyroid peroxidase antibodies; TgAb, antithyroglobulin antibodies; hsCRP, high sensitivity C reactive protein; T1-L-Thyroid, The T1-mapping values of the left thyroid lobar; T1-R-Thyroid, The T1-mapping values of the right thyroid lobar; T1-Thyroid, the averages of T1-mapping values of the left and right thyroid lobars; P_1_, the hypothyroidism group before levothyroxine treatment vs. the control group, analyzed by independent Student's t-test or the Mann–Whitney U-test; P_2_, comparison of parameters in the hypothyroidism group after levothyroxine treatment, analyzed by a two-tailed paired t-test or Wilcoxon rank test; P_3_, the hypothyroidism group after levothyroxine treatment vs. the control group, analyzed by independent Student's t-test or the Mann–Whitney U-test*.

### Influence of Levothyroxine Treatment on Clinical Parameters and Thyroid T1-Mapping Values

**Figure 1:**
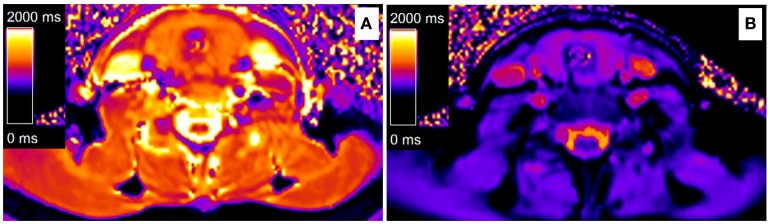
Color maps of thyroid T1-mapping based on a Modified look-locker inversion-recovery sequence. **(A)** A hypothyroid AIT patient before levothyroxine treatment (T1 = 1,430 ms), **(B)** A hypothyroid AIT patient after levothyroxine treatment (T1 = 978 ms). **(A,B)** came from the same patient.

**Figure 2:**
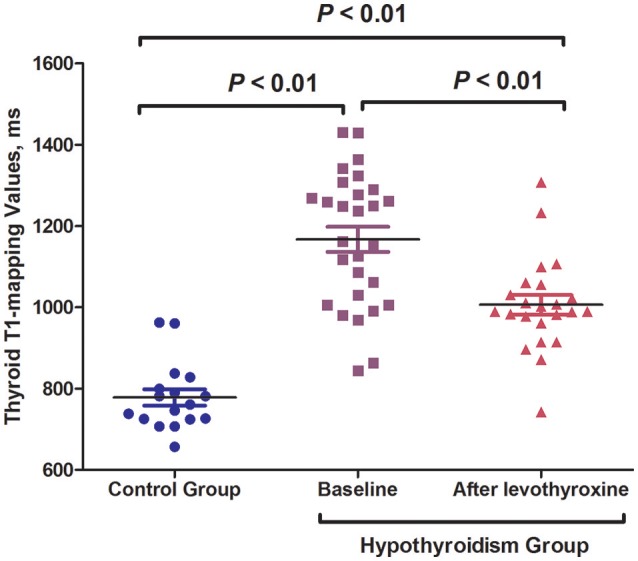
Levothyroxine treatment significantly decreased the thyroid T1-mapping values of hypothyroid AIT patients.

All the hypothyroid patients completed the follow-up of this study. No serious adverse events were recorded during the levothyroxine treatment. The euthyroid state was achieved with a mean dose of 109.4 μg/d in 12.8 ± 3.2 weeks. The hypothyroid patients who underwent levothyroxine treatment had FT3, FT4, and TSH levels that were similar to those of the control subjects (Table 1). After levothyroxine treatment, the levels of body weight and BMI significantly decreased in the hypothyroidism group (24.5 ± 4.1 vs. 26.0 ± 3.9 kg/m^2^, *P* < 0.01), but the BMI levels after levothyroxine treatment were still higher than that in the control group (24.5 ± 4.1 vs. 22.0 ± 3.2 kg/m^2^, *P* < 0.05). Moreover, the levothyroxine treatment significantly decreased the TPOAb and TgAb levels of the hypothyroid patients [TPOAb: 257.6 (23.9–960.6) vs. 1287.4 (12.6–2000.0) IU/mL, *P* < 0.01; TgAb: 53.54 (9.58–386.2) vs. 103.9 (34.2–1596.8) IU/mL, *P* < 0.05; Table 1]. The hsCRP levels showed a descending tendency following levothyroxine treatment, although there was no statistical difference (Table 1). Notably, the thyroid T1-mapping values significantly decreased in the hypothyroid AIT patients following the levothyroxine treatment (1006.3 ± 114.6 vs. 1167.2 ± 163.2 ms, *P* < 0.01), but were still higher than those in the control subjects (1006.3 ± 114.6 vs. 779.6 ± 83.8 ms, *P* < 0.01; Table 1, Figures 1, 2).

## Discussion

In the present study, thyroid T1-mapping values were higher in the hypothyroid AIT patients than in the healthy controls. And levothyroxine treatment significantly decreased the thyroid T1-mapping values of the hypothyroid AIT patients. Meanwhile, reduced levels of TPOAb and TgAb were observed in the AIT patients after levothyroxine treatment. In the hypothyroid AIT patients, the hsCRP levels showed a descending tendency following levothyroxine treatment.

AIT is the most frequent cause of hypothyroidism (2, 4). The typical thyroid lesions of AIT patients include diffuse lymphocytic infiltration, fibrosis, and epithelial cell destruction (14). Magnetic resonance T1-mapping is a parametric reconstructed image analyzed using a MOLLI sequence (7–10). T1-mapping values were positively associated with the degree of histological fibrosis (9, 11). In addition, T1-mapping values better reflected myocardial edema than conventional T2-weighted values because of the higher sensitivity to water (12, 13). Our previous studies have demonstrated that TSH levels positively correlated with T1-mapping values, and the thyroid T1-mapping values quantitatively evaluate the degree of thyroid destruction in AIT patients (7). In the present study, thyroid T1-mapping values were significantly higher in the AIT patients than in the healthy controls, and levothyroxine replacement significantly decreased the thyroid T1-mapping values of AIT patients. Thus, these results might suggest that levothyroxine treatment alleviates thyroid destruction in hypothyroid AIT patients. Because magnetic resonance T1-mapping values reflect the levels of tissue fibrosis and edema, the decreased thyroid T1-mapping values following levothyroxine replacement suggested that the levels of fibrosis and edema were relieved in the thyroid gland of AIT patients. However, it was difficult for an image examination to identify how much of the decrease in T1-mapping values was due to the amelioration of edema or fibrosis. In AIT patients, infiltrated lymphocytes lead to inflammatory edema, and subsequent tissue fibrosis in thyroid (4). It is unlikely that a short-term levothyroxine could reduce thyroid fibrosis.

AIT is a complex disease mainly mediated by a cellular autoimmune response, while an evident humoral autoimmune response is also observed (4). The CD8+ cytotoxic T cells directly destroy thyroid cells and cause thyroid edema by inducing cell necrosis or apoptosis (16, 17). Activated CD4+ T lymphocytes impair thyroid cells by producing cytokines, such as tumor necrosis factor-α (TNF-α) and interleukin-1 (IL-1), and further induce more lymphocyte infiltration (16, 17). Nowadays, both T regulatory (Treg) and B regulatory (Breg) lymphocytes were being increasingly recognized as key elements in the pathogenesis of AIT (18, 19). Treg cells play a vital role in immune tolerance by eliminating autoreactive clones, whereas Breg cells inhibit pro-inflammatory response mainly by secreting interleukin-10 (IL-10) (18, 19). The dysfunction of Treg and Breg cells caused the preferred differentiation of lymphocytes toward pro-inflammatory cell subtypes, such as Th1 and Th17 cells, and further results in the increased production of inflammatory cytokines and an inflammatory state (18–20). The present study showed that the hypothyroid AIT patients had increased hsCRP levels when compared with the control group, and the hsCRP levels showed a descending tendency following levothyroxine treatment. Since the hsCRP was commonly regarded as a maker for inflammatory state of the body, the decreased hsCRP levels following levothyroxine treatment might suggest the alleviation of immune inflammatory response. Notably, there are some disputes which should be clarified (21, 22). Hypothyroidism is associated with a chronic inflammatory state (21, 23–26). TSH directly induces the secretion of interleukin-6 (IL-6) in 3T3-L1 adipocytes and TNF-α in bone marrow cells *in vitro* (23, 24). Moreover, administration of TSH increased the serum levels of inflammatory factors, including IL-6 and TNF-α, and led to a chronic inflammatory status in humans (25, 26). Also, obesity and insulin resistance are possible confounders in determining the increase in hsCRP levels in hypothyroid AIT patients (27). Thus, levothyroxine treatment restored thyroid function, reduced TSH levels, improved metabolic disorders, and decreased body weight in patients with hypothyroidism, which might be involved in the reduced hsCRP levels. However, in the euthyroid AIT patients, levothyroxine treatment still significantly decreased the inflammatory factors and the lymphocytes in thyroid (28, 29). Moreover, levothyroxine reduced the expression and release of inflammatory factors, including TNF-α, interleukin-1β (IL-1β), IL-6, and monocyte chemoattractant protein-1 in the monocytes of the euthyroid AIT patients (29). TPOAb and TgAb are the two important antibodies of humoral immunity in AIT. Both TPOAb and TgAb are mainly produced by B lymphocytes infiltrated in the thyroid (30, 31). And the TPOAb levels were positively associated with the infiltrated lymphocyte count in the thyroid gland (30). The present study showed that levothyroxine treatment significantly decreased the TPOAb and TgAb levels of the hypothyroid patients. These results are also supported by some previous studies. Levothyroxine treatment significantly reduced the TPOAb levels in both hypothyroid and euthyroid patients with AIT (28, 29, 32). On the one hand, the decrease in TPOAb level is probably due to increased clearance of TPOAb following levothyroxine treatment. On the other hand, the decreased TPOAb level also might suggest the alleviated humoral immunity.

The present study is the first one focused on the effect of levothyroxine on thyroid destruction in hypothyroid AIT patients using thyroid T1-mapping technique. Our study found that in addition to ameliorating metabolic disorders and restoring the functions of heart and skeletal muscle, levothyroxine treatment also alleviates thyroid destruction in hypothyroid AIT patients. These results might suggest that early treatment has potential to delay disease progression for the hypothyroid AIT patients. However, some limitations of the present study should be mentioned. First, this study was a single-center study with a relatively small sample size, which might have introduced some confounders that influenced the results. Second, since magnetic resonance T1-mapping values reflect the levels of tissue fibrosis and edema, it may be challenging to distinguish how much of the decrease in T1-mapping values was due to the amelioration of edema or fibrosis. Then, the follow-up period was short, and we observed only short-term effects of levothyroxine treatment on thyroid T1-mapping values in AIT patients with hypothyroidism. Further long-term and large-scale studies, especially in subclinical hypothyroid AIT patients, are needed to confirm the results we reported.

In conclusion, hypothyroid AIT patients had increased thyroid T1-mapping values, and the thyroid T1-mapping values were significantly decreased after levothyroxine treatment. These results might suggest that levothyroxine treatment alleviates thyroid destruction in hypothyroid AIT patients.

## Data Availability

All datasets generated for this study are included in the manuscript and/or the supplementary files.

## Author Contributions

JL and GW conceived and designed the experiments. JL, ZC, ML,YJ and ZY performed the experiments. JL and GW analyzed the data. JL wrote the paper.

### Conflict of Interest Statement

The authors declare that the research was conducted in the absence of any commercial or financial relationships that could be construed as a potential conflict of interest.
